# Relationship Between Rennet Coagulation Properties of Milk, Cheese-Making Losses, and Cheese Yield in Manufacture of Parmigiano Reggiano PDO Cheese

**DOI:** 10.3390/foods15030428

**Published:** 2026-01-24

**Authors:** Piero Franceschi, Davide Barbanti, Paolo Formaggioni, Cristina Scotti, Paola Giambiasi, Francesca Martuzzi

**Affiliations:** 1 Department of Food and Drug, University of Parma, Parco Area delle Scienze, 27/A, 43124 Parma, Italy; davide.barbanti@unipr.it (D.B.); francesca.martuzzi@unipr.it (F.M.); 2 Centro Lattiero Caseario ed Agroalimentare, Strada dei Mercati 22, 43126 Parma, Italy; c.scotti@clca.it (C.S.); p.giambiasi@clca.it (P.G.)

**Keywords:** milk composition, rennet coagulation properties, cheese yield, cheese-making losses, Parmigiano Reggiano cheese

## Abstract

The aim of this study was to assess the influence of milk’s rennet coagulation properties (RCPs) on cheese yield and cheese-making losses in the production of Parmigiano Reggiano PDO cheese. Higher contents of citric acid (181.10 vs. 172.13 vs. 166.47 mg/100 g) and phosphorus (95.02 vs. 91.14 vs. 88.78 mg/100 g) in milk with optimal and sub-optimal RCPs, compared to milk with poor RCPs, respectively, positively affect the acidity of the milk, lowering the pH values (6.68 vs. 6.70 vs. 6.72, respectively), which results in a faster reaction between chymosin and casein and consequently a reduced time of milk coagulation. The lower values of curd firming time and the higher values of curd firmness, strength to cut (68.97 vs. 64.43 vs. 44.38 g), and strength to compression (31.48 vs. 30.49 vs. 25.70 g) for milk with optimal and sub-optimal coagulation, compared to milk with poor coagulation, result in a higher stress resistance across the technological steps of the cheese-making process, leading to lower fat losses (14.23 vs. 15.48 vs. 16.72%) in the whey and a higher cheese yield (8.79 vs. 8.56 vs. 8.08 kg/100 kg).

## 1. Introduction

In the production of hard cooked cheeses like Parmigiano Reggiano PDO, the main aim of the cheese-making process is the recovery of all of the casein and most of the fat matter in the milk; this is achieved by means of coagulation. In this process, chymosin of rennet is used to hydrolyse the glycomacropeptide of k-casein in casein micelles, which leads to the formation of curd [1]. Curd is basically a paracasein net that, during its formation, entraps fat globules and a fraction of milk whey. After its formation, this casein net tends to contract, and during this contraction process, fat globules are retained within the curd, while milk whey, with lactose, minerals, and whey protein, is expelled from the curd [2,3].

For these reasons, the ability of milk to give rise to a curd of good quality, after addition of the rennet, is one of the most important requirements in the majority of cheese production processes [4]. Indeed, milk with optimal rennet coagulation characteristics produces curds with better rheological properties and is characterised by optimal syneresis ability, with positive repercussions on all phases of the cheese-making process and producing better quality cheese [1,2,5]. On the contrary, curds obtained from milk with poor rennet coagulation properties are more friable and poorly retain fat globules, resulting in an increase in cheese-making losses across the technological steps of cheese-making [6,7]. Furthermore, since these curds are often characterised by poor syneresis properties, they also show incomplete and non-homogeneous drainage of the whey, accompanied by an increase in spoilage bacterial activities that result in an increase in structural defects in cheese wheels across all stages of the ripening process [8,9,10].

**Figure 1:**
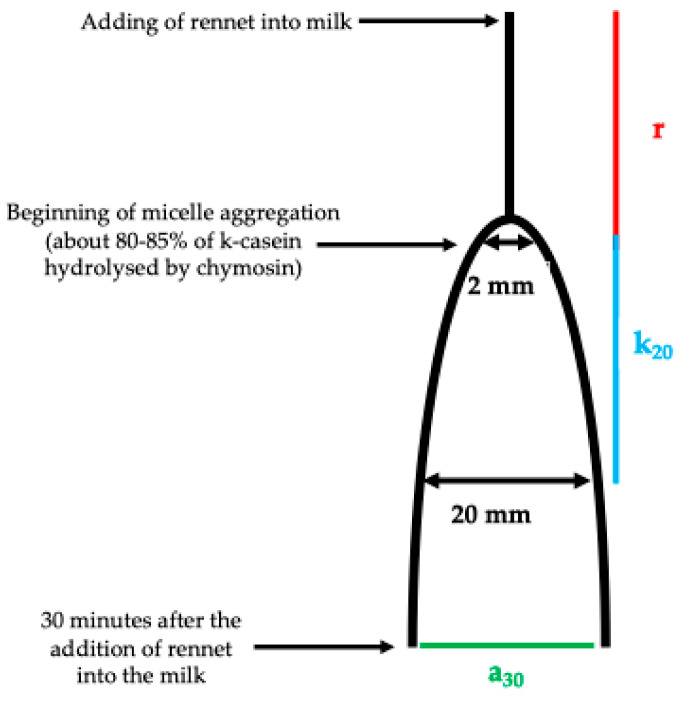
Bell-shaped graph obtained by lactodynamographic test, where “r” is clotting time, measured in minutes, “k_20_” is curd firming time, measured in minutes, and “a_30_” is curd firmness, measured in millimetres at 30 min after addition of rennet.

The lactodynamographic (LDG) test is one of the most widespread methods used to analyse the rennet coagulation properties of milk [5,11,12]. This technique detects the change in viscosity in a milk sample during gel formation induced in milk by rennet [5,11]. Modifications of milk viscosity are translated by the device into a graph, namely a bell-shaped diagram (Figure 1), from which the three main parameters of rennet coagulation properties can be derived [5,11].

Clotting time (r) is the time from the addition of rennet into the milk to the beginning of coagulation; curd firming time (k_20_) is the time from the start of coagulation to reaching a graph with a 20 mm width; curd firmness (a_30_) is the width, expressed in millimetres, of the graph at 30 min after the addition of the rennet.

In many dairy sectors, the lactodynamographic test is used in the milk payment system [13], and currently, in the Parmigiano Reggiano area, this test is performed on herd milk samples twice a month. Its values, divided into classes based on the clotting time and curd firmness, are used to establish the monetary value of milk in the milk quality payment system [14].

Furthermore, many studies report that milk with better rennet coagulation properties is associated to a higher cheese yield. For example, Ng-Kwai-Hang et al. [15] recorded that the rennet coagulation properties of milk are correlated with cheese yield.

Moreover, more recently, Bittante et al. [12] proposed that the milk coagulation properties can be considered a good indicator of the cheese yield ability of milk, while Pretto et al. [16] reported that milk coagulation properties could be indicators of cheese-making efficiency, because milk with higher values of curd firmness was characterised by a higher cheese yield when compared to milk with lower values of curd firmness.

On the other hand, it should be acknowledged that there exist studies which did not find any correlation between the rennet coagulation properties of milk, its cheese yield ability, and its cheese-making efficiency.

For example, Wedholm et al. [17] did not find a significant difference in cheese yield ability across cheese-making processes using milk characterised by optimal rennet coagulation properties and milk with poor rennet coagulation properties. More recently, Bonfatti et al. [13] concluded that the differences in milk rennet coagulation properties do not affect milk’s cheese yield ability.

Overall, the contrasting conclusions of these studies could stem from differences in the conditions of cheese-making processes and in the methods used to determine the rennet coagulation properties.

Indeed, methods used for the determination of the rennet coagulation properties, representing laboratory analyses, do not involve steps typical of the cheese-making process, like milk heating, milk acidification with whey starter, cutting of the curd, and cooking of the curd.

In addition, the duration and rendering mode of these technological treatments vary considerably among different types of cheese and they strongly affect cheese yield and cheese-making efficiency.

Due to the economic relevance of Parmigiano Reggiano PDO cheese and the influence of rennet coagulation parameters in determining the quality and economic value of milk at the farm, studying the relationships between the milk rennet coagulation properties and cheese yield in the production of Parmigiano Reggiano cheese can be of relevance. Indeed, during 2023, Parmigiano Reggiano cheese production was about 161 thousand tons, with over 2 million tons of milk processed in cheese, and this involved about 15.6% of Italian milk produced.

Since the lactodynamographic test is used in the milk payment system for Parmigiano Reggiano production, highlighting how the milk rennet coagulation properties affect cheese yield in field conditions could improve the milk payment system and strengthen the Parmigiano Reggiano sector.

For these reasons, the aim of this study was to assess the influence of the milk rennet coagulation properties on cheese yield in the milk cheese-making process in Parmigiano Reggiano, emphasizing the relationships between milk chemical composition, milk rennet coagulation properties, cheese yield, and cheese-making losses of milk compounds into whey at the end of the cheese-making process.

## 2. Materials and Methods

### 2.1. Parmigiano Reggiano Cheese-Making

In full compliance with the Parmigiano Reggiano cheese production regulation [18], cows were milked twice a day, in the early morning and in the evening. After each milking, the milk, cooled at 18 °C, was collected and delivered to the cheese factory.

The milk of evening milking was placed in the creaming tank immediately after its arrival in the cheese factory, and the creaming process occurred overnight.

The morning after, partially skimmed milk (fat content of approximately 1.6 g/100 g) was extracted via draining from the bottom of the tank and placed in the vat. Then, full-cream milk of the morning milking was added to obtain the vat milk. In this phase of the process, the amounts of both partially skimmed milk and full-cream milk were regulated to obtain a fat-to-casein ratio in the vat milk of about 1.10.

Afterwards, in the vat milk, a natural whey starter, produced by natural fermentation of the whey remaining after the cheese-making process of the previous day, was added (about 1.5 kg/100 kg of milk), and this mixture was heated at 33 °C. Then, 3 g of calf rennet per 100 kg of milk was added to the mixture. Rennet was previously prepared by dissolving the rennet powder (strength of 1:120,000; Caglio Bellucci Srl, Modena, Italy) in about 3 litres of water.

Coagulation occurred from 12 to 15 min after the addition of the rennet into the vat milk, and two minutes after coagulation, the curd was broken in small granules (approximately 5 millimetres of diameter) by stirring.

Immediately after the end of the breaking operation, the curd was cooked, raising the temperature of the vat content to 55 °C. Cooking took a total of 15 min, and during the cooking phase, stirring was maintained.

After the cooking phase, stirring was stopped and the cheese granules were left to deposit and blend at the bottom of the vat for one hour.

The cheese was then taken out from the bottom of the vat and divided into two cheese wheels that were placed in cylindrical moulds for 48 h. Since the content of the vat has undergone a cooking process, the whey that remains in the vat after the extraction of the cheese is named cooked whey.

Moreover, during the 48 h in which the cheese wheels were held in the moulds, they partially cooled and, during the cooling, acidified due to the fermentation of lactose by thermophilic lactic acid bacteria.

After cooling, the cheese wheels were salted in brine for 20 days and then placed into the ripening room for at least 12 months. At the end of the process, Parmigiano Reggiano cheese wheels have a cylindrical shape with a diameter of about 45 cm, a height of approximately 26 cm, and a weight of about 40 kg.

### 2.2. Experimental Design

The experimental design is summarized in Table 1. In 2 years, across 10 cheese factories located in the provinces of Parma and Reggio Emilia, a total of 60 cheese-making processes using milk for Parmigiano Reggiano cheese were performed during 20 trials (2 trials in each cheese factory). Parmigiano Reggiano cheese-making processes were carried out in field conditions and only Italian Friesian cow milk was processed.

In each trial, 3 cheese-making processes were performed in parallel: the first with vat milk characterised by optimal rennet coagulation properties; the second with vat milk characterised by sub-optimal rennet coagulation properties; and the third with vat milk characterised by poor rennet coagulation properties.

Optimal, sub-optimal, and poor rennet coagulation property classes were defined based on the values of clotting time and curd firmness of V-milk, as reported in Table 2.

In brief, the lactodynamograms obtained by the rennet coagulation analysis were classified in different lactodynamographic types, indicated with capital letters from “A” to “EF”, in agreement with Malacarne et al. [4].

Afterward, the lactodynamographic types were grouped, according to Franceschi et al. [14], into three classes:

- Optimal (optimal RCP), including lactodynamographic types A, B, and AB (20 cheese-making trials);
- Sub-optimal (sub-optimal RCP), including lactodynamographic types C, AE, EA, AD, and CC (20 cheese-making trials);
- Poor (poor RCP), including lactodynamographic types D, E, and EF (20 cheese-making trials).

In addition, since the scientific literature [3,16,19] suggests that in the production of cheese with raw milk, milk contents of protein and casein strongly affect cheese yield, to equalise the other sources of variation, the bulk milks used for cheese-making in the three experiments in each trial were selected with similar contents of crude protein and casein and with similar and moderate values of somatic cells and total bacterial counts.

Moreover, since in the production of cheese with partially skimmed milk, the fat-to-casein ratio of vat milk affects both cheese yield and cheese-making losses of fat [7,19,20], the vat milk in the trials was standardised at a fat-to-casein ratio of about 1.1.

### 2.3. Sample Collection and Sampling Procedure

Furthermore, for each cheese-making process of each experimental trial, the following sampling was carried out:

- A sample of whole milk of the evening milking (W-milk), sampled directly from the creaming tank, before the start of the natural creaming process.
- A sample of vat milk (V-milk), consisting of a mixture of the partially skimmed milk of the evening milking, obtained by natural creaming, and the whole milk of the morning milking. Sampling was performed directly in the vat milk at the beginning of the cheese-making process, before the addition of the whey starter.
- A sample of residual cooked whey (C-whey), taken after the extraction of the cheese from the vat milk and after stirring the cooked whey for 5 min.

The samples W-milk, V-milk, and C-whey were cooled at 5 °C, transported to the laboratory, and immediately analysed.

### 2.4. Analytical Methods

The previously described samples were submitted to analysis to determine the following parameters.

Lactose in V-milk, fat in W-milk, V-milk, and C-whey, and crude protein and casein in W-milk were assessed using mid-infrared spectrometry [21] with Milko-Scan FT-plus (Foss Electric, DK-3400 Hillerød Denmark).

Somatic cell count was determined in W-milk and V-milk samples by using a fluoride-opto-electronic method [22] with Fossomatic FC (Foss Electric, see above), while the total bacterial count was determined in W-milk and V-milk by using the flow cytometry method [23] with BactoScan FC (Foss Electric, see above).

Furthermore, the nitrogen fractions total N (TN), non-casein N (NCN), and non-protein N (NPN) were also determined in V-milk by using the Kjeldahl method [24,25,26], and according to the difference between total N and non-casein N (TN-NCN) values, casein N (CN) was calculated. Starting from these values, as described by Lynch et al. [27], the values of crude protein (TN × 6.38/1000), whey protein (NCN × 6.38/1000), casein (CN × 6.38/1000), casein number (CN × 100/TN), and NPN × 6.38 (NPN × 6.38/1000) were calculated. By using the Kjeldahl method, the total nitrogen content of C-whey samples [24] was determined, and similarly to what was described for milk, the value of crude protein (TN × 6.38/1000) was calculated.

In V-milk samples, the values of pH and titratable acidity were also assessed, the first by using a potentiometer and the second according to the Soxhlet–Henkel method [28] via titration of 50 mL of milk with 0.25 N sodium hydroxide.

Dry matter and ash were determined both in V-milk and in C-whey by drying at 102 °C [29] and by calcination in muffle at 530 °C [30]. Starting from the ashes of V-milk and of C-whey samples dissolved in hydrochloric acid 2 N, the total content of calcium, phosphorus, and magnesium was determined.

In particular, calcium and magnesium were assessed by Atomic Absorption Spectroscopy [31], while phosphorus content was determined by using the colorimetric method of Allen [32].

In addition to these minerals, chloride content and citric acid were assessed in V-milk samples, the first by titration with silver nitrate [33] and the second by using a chromatographic method [34].

In V-milk samples, the rennet coagulation parameters were determined, including clotting time, curd firming time, and curd firmness, by using Formagraph (Foss Electric, see above), operating at 35 °C, according to McMahon and Brown [11]. In brief, 0.2 mL of rennet (strength of 1:19,000 of Chr. Hansen, I-20094 Corsico, MI, Italy) was added to 10 mL milk and, in the graph produced by the Formagraph, the rennet coagulation parameters were measured. Moreover, the strength to cut and strength to compression of the curd were also determined in V-milk using the Gel Tester of Marine Colloids (Marine Colloids Inc., Springfield, NJ, 07081, USA), testing the curd 30 min after the beginning of coagulation [4].

In addition, for each cheese-making process, the V-milk weight and the cheese weight at 24 h after the extraction from the vat were measured, and from these two parameters, the cheese yield (expressed as kg of cheese for each 100 kg of milk) was calculated, according to Franceschi et al. [20], as follows:

CY = [CW (Kg)] × 100/MW (kg)(1)

where ACY is the actual cheese yield of the V-milk, CW is the weight of cheese obtained from the V-milk, and MW is the weight of the V-milk used for the cheese-making process.

In addition, the estimated cheese-making losses (ECLs) for protein, fat, calcium, phosphorus, and magnesium were calculated according to Franceschi et al. [7], as reported below:

ECL = N^th^C-whey × 100/N^th^V-milk (2)

where ECL is the value of the estimated cheese-making losses, expressed as a percentage; N^th^C-whey is the content of the N^th^ constituent in the C-whey, expressed as g/100 g for protein and fat and as mg/100 g for Ca, P, and Mg; and N^th^V-milk is the content of the N^th^ constituent in the V-milk, with these values also expressed as g/100 g for protein and fat and as mg/100 g for Ca, P, and Mg.

Finally, the somatic cell count of each sample was log-linearised before statistical analysis; furthermore, based on the values of casein and fat, the fat-to-casein ratio in the V-milk samples was calculated.

### 2.5. Statistical Analysis

Starting from the classification of optimal, sub-optimal, and poor RCP classes, data were analysed by one-way analysis of variance and the least square means were calculated with the general linear model procedure of software SPSS 29 (IBM SPSS Statistics, 10504-1722, Armonk, New York, NY, USA), according to the following general linear model:

Y_ijk_ = µ + L_i_ + T_j_ + ε_ijk_ (3)

where:

Y_ijk_ = dependent variable; µ = overall mean; L_i_ = effect of the class of lactodynamographic type (optimal, discrete, and poor), divided into 3 levels, (i = 1,…, 3); T_j_ = effect of the trial, divided into 20 levels (j = 1,…, 20); ε_ijk_ = residual error.

Furthermore, the significance of the differences among the classes of the lactodynamographic types were tested by using the Bonferroni post hoc method.

In addition, the Pearson correlation coefficients of milk rennet coagulation properties and rheological parameters with the values of cheese yield and the estimated cheese-making losses were calculated. The Pearson correlation coefficients of milk mineral contents with milk rennet coagulation properties and rheological parameters were also calculated.

## 3. Results

In Table 3, the chemical composition parameters and the somatic cell count of whole milk from the evening milking (W-milk) with optimal, sub-optimal, and poor rennet coagulation properties are shown.

Among the parameters reported in Table 3, only milk fat content showed significant differences among the classes with *p* ≤ 0.05, and it was higher in W-milk with optimal and sub-optimal RCPs than in W-milk with poor RCPs. The difference in the W-milk fat content between milk with sub-optimal RCPs and poor RCPs was 0.1 g/100 g and can be considered negligible.

In Table 4, the chemical composition parameters, the mineral contents, and the somatic cell count of vat milk (V-milk) with optimal, sub-optimal, and poor rennet coagulation properties are shown.

The V-milk content of lactose and phosphorus showed significant differences across the three classes with *p* ≤ 0.001, while calcium content was significantly different with *p* ≤ 0.01. Furthermore, V-milk contents of citric acid, chloride, and magnesium and somatic cell count showed significant differences among optimal, sub-optimal, and poor RCP classes with *p* ≤ 0.05.

Lactose and magnesium were higher in optimal-RCP and sub-optimal-RCP milk than in poor-RCP milk, while calcium, phosphorus, and citric acid were higher in optimal-RCP milk and lower in poor-RCP milk, with intermediate values in sub-optimal-RCP milk.

On the other hand, milk chloride contents were lower in optimal-RCP and sub-optimal-RCP milk and higher in poor-RCP milk, while the somatic cell count was higher in milk with optimal RCPs and poor RCPs than in milk with sub-optimal RCPs.

Table 5 reports the physico-chemical characteristics, rennet coagulation properties, and curd rheological parameters of vat milk with optimal, sub-optimal, and poor rennet coagulation properties.

Since samples were divided according to clotting time and curd firmness, their values showed significant differences with *p* ≤ 0.001 among the three classes.

In addition, the value of titratable acidity showed significant differences, with *p* ≤ 0.001, among optimal, sub-optimal, and poor RCP classes.

Moreover, curd strength to cut and curd strength to compression showed significant differences among optimal, sub-optimal, and poor RCP classes with *p* ≤ 0.01, while the V-milk pH values and the curd firming time showed significant differences among optimal, sub-optimal, and poor RCP classes with *p* ≤ 0.05.

Titratable acidity was higher in optimal-RCP milk and lower in poor-RCP milk, with intermediate values in sub-optimal-RCP milk, while the pH value and curd firming time were lower in optimal-RCP milk and higher in poor-RCP milk, with intermediate values in sub-optimal-RCP milk.

Finally, the strength to cut and strength to compression of the curds were higher in milk with optimal RCPs and sub-optimal RCPs than in milk with poor RCPs.

Table 6 reports the actual cheese yield values and the cheese-making loss parameters of vat milk with optimal, sub-optimal, and poor rennet coagulation properties.

The actual cheese yield and the fat losses showed significant differences among optimal, sub-optimal, and poor RCP classes, the first with *p* ≤ 0.01 and the second with *p* ≤ 0.05.

The actual cheese yield was higher in optimal RCP milk and lower in poor-RCP milk, with intermediate values in sub-optimal-RCP milk, while the cheese-making losses of fat were lower in optimal-RCP milk and higher in poor-RCP milk, with intermediate values in sub-optimal-RCP milk.

In Table 7, the Pearson correlation coefficients of milk mineral contents with milk rennet coagulation properties and rheological parameters are reported; meanwhile, in Table 8, the Pearson correlation coefficients of rheological parameters with milk rennet coagulation properties and rheological parameters are reported.

Clotting time was negatively correlated with milk contents of calcium, phosphorus, and citric acid (*p* ≤ 0.01). Curd firmness showed a positive correlation with the same milk minerals, but with *p* ≤ 0.001 in relation to milk calcium content and with *p* ≤ 0.05 in relation to the contents of phosphorus and citric acid. Furthermore, curd firming time was negatively correlated with the calcium and phosphorus content of milk (*p* ≤ 0.01).

The milk contents of calcium and phosphorus were positively correlated with curd strength to cut and curd strength to compression, both with *p* ≤ 0.001, and these two parameters showed correlations with both curd firming time and curd firmness as follows:

- Strength to cut was negatively correlated with curd firming time (*p* ≤ 0.001) and positively correlated with curd firmness (*p* ≤ 0.01);
- Strength to compression showed a negative correlation with curd firming time and a positive correlation with curd firmness, both with *p* ≤ 0.001.

In Table 9, the Pearson correlation coefficients of actual cheese yield and protein and fat losses with milk rennet coagulation properties and rheological parameters are reported.

The actual cheese yield was negatively correlated with curd firming time and positively correlated with curd firmness (both with *p* ≤ 0.01); it was also positively correlated (*p* ≤ 0.001) with values of strength to cut and strength to compression.

Furthermore, the actual cheese yield was negatively correlated with the values of fat loss with *p* ≤ 0.05.

Finally, fat loss was positively correlated (*p* ≤ 0.001) with curd firming time and negatively correlated (*p* ≤ 0.05) with curd firmness, strength to cut, and strength to compression.

## 4. Discussion

### 4.1. Chemical Composition, Physico-Chemical Properties, and Rheological Parameters of Milk

In general, milk RCPs depend on milk casein content and milk acidity; many studies report a negative correlation between milk casein content and clotting time and a positive correlation between milk pH value and clotting time [35,36]. In particular, an decrease in milk pH increases both the activity of chymosin and the amount of diffusible ionic calcium, with positive repercussions on the primary and secondary phases of the milk coagulation process [1,4].

Therefore, since the casein content of the V-milk did not show significant differences among optimal RCP, sub-optimal RCP, and poor RCP groups, as planned in the experimental design, the differences in the RCPs of the milk of the three classes were mainly due to their different milk pH values.

The pH value of milk, in turn, depends mainly on the phosphorus and citric acid contents of the milk and the balance between the calcium phosphate in solution and the colloidal calcium phosphate contained as a component of the casein micelle [4].

It is important to highlight that these elements in milk are mainly divided into two different phases [4,37,38,39,40]. About two-thirds of calcium and half of the content of phosphorus are in the colloidal phase as a constituent of the casein micelles, while the remaining parts are in solution [4,37,38].

Inside the casein micelles, phosphorus and calcium can be covalently bound to the serine of caseins (phosphoserine) or as salt calcium phosphate [4,37,38].

On the other hand, the part of salt dissolved in solution is present as dibasic calcium phosphate [4,39,40].

Dibasic calcium phosphate is a weak acid that, with its conjugated salt with calcium (calcium phosphate), constitutes a buffer.

This buffer stabilizes the milk pH value, from 6.66 to over 6.70, depending on the concentrations of the two salts [4,38,41].

Moreover, the contents of calcium, phosphorus, and magnesium are in agreement with those reported by Malacarne et al. [4] for the sub-optimal-RCP milk (113.12; 89.75; 9.54 mg/100 g, respectively) and poor-RCP milk (111.88; 88.75; 9.47 mg/100 g, respectively), while the contents of calcium and phosphorus of optimal-RCP milk are lower compared with those reported by Malacarne et al. [4] (129.54 and 106.59 mg/100 g, respectively).

Therefore, the differences in the contents of citric acid, phosphorus, and calcium of the optimal RCP, sub-optimal RCP and poor RCP groups affect the differences in their pH values, giving rise to different clotting times, curd firming times, and curd firmness. This observation is confirmed by the differences in the contents of calcium, phosphorus, and citric acid recorded among optimal, sub-optimal, and poor RCP groups, as well as by the negative Pearson correlation coefficients recorded between milk clotting time and the milk contents of calcium, phosphorus, and citric acid.

The differences in the milk mineral contents among the three classes were probably due to small and sparsely diffused inflammation processes in the cow udders on the farms. This observation was confirmed by the differences in the chloride content among the three classes; chloride was higher in poor-RCP milk than in the other two classes. Indeed, in the literature, many authors have reported that during the inflammation processes of the mammary gland, there is a diffusion of sodium chloride from the blood to the milk [42,43].

Moreover, since lactose synthesis occurs in the Golgi apparatus of the mammary gland cells, and, after, lactose is secreted by vesicles from the alveolar cells in the alveolar lumen [44,45,46], a decreasing in the secretory activity of the mammary cells due to the inflammation process negatively affects milk lactose content [43,47].

In severe cases, when udder inflammation is clinical, the inflammatory condition is also associated with a general decrease in milk casein content [48,49] and with an increase in the leukocyte number in milk, with a consequent increase in somatic cell count [38,43]. Moreover, since cheese is essentially constituted by a casein net that, during its formation, incorporates fatty globules [50,51], the general lowering of casein values results in a decrease in cheese yield [52,53,54]. The negative effect of somatic cell count on cheese yield is attested in the production of hard cooked cheeses like Grana Padano [16] and Parmigiano Reggiano [43].

Nevertheless, Summer et al. [43], in their study aiming to investigate the effect of different levels of milk somatic cell counts on Parmigiano Reggiano cheese yield, recorded that the reduction in cheese yield starts at 300 10^3^ cells/mL. Overall, in the present study, the value of whole-milk somatic cell count was always under 300 10^3^ cells/mL, and thus, the inflammation process in cow udders can be considered a small entity that does not cause differences in cheese yield among the three classes.

### 4.2. Curd Rheological Properties, Cheese Yield, and Cheese-Making Losses

V-milk with optimal and sub-optimal RCPs was characterised by curds with higher values of both strength to cut and strength to compression compared to poor-RCP V-milk.

In general, the rennet coagulation process of bovine casein is divided into two phases [55,56,57]. In the primary phase, chymosin, which is the main enzyme of rennet, hydrolyses k-casein by splitting the glycomacropeptide from paracasein [1,37]. When about 80% of the k-casein in a micelle has been hydrolysed, the secondary phase begins [4,37,56]. In the secondary phase, paracasein micelles aggregate together through calcium phosphate bonds formed by calcium ions in solution [56,57].

Because chymosin is an acid protease, the lower the pH of the milk, the faster the primary phase will be and thus the coagulation time will be shorter. In this regard, the milk contents of citrate and calcium phosphate affect the pH value, lowering it and accelerating the primary phase of the coagulation process, resulting in a decrease in clotting time [1,4]. Moreover, higher contents of calcium in solution result in a faster secondary phase, with more rapid aggregation of casein micelles to form the curd, resulting in a lower curd firming time and a higher curd firmness [4,56].

Therefore, the values of strength to cut and strength to compression mainly depend on curd firming time and curd firmness, as was confirmed by many correlations observed in this study. In fact, a negative correlation was found between curd firming time and curd firmness of V-milk and the values of the strength to cut and strength to compression of curd obtained using this milk. Moreover, a positive correlation was found between the contents of calcium and phosphorus in V-milk and the values of strength to cut and strength to compression of its curd.

V-milk with optimal RCPs was characterised, when compared to that with poor RCPs, by a higher value of cheese yield and a lower value of fat loss. This is due to the better rheological properties of the milk of the first group compared to the second group. In fact, V-milk with optimal RCPs, because of its better rennet coagulation properties, gives rise to a curd with higher strength to cut and higher strength to compression during the rennet coagulation process. These better physico-mechanical properties of the curd allow it to better withstand the mechanical stresses of curd breaking and cooking during cheese-making. As a result, the curd of V-milk with optimal RCPs can better retain fat globules during the cheese-making process compared to the curd of V-milk with poor RCPs, and, therefore, the curd of V-milk with optimal RCPs has lower values of fat losses than the curd from V-milk with poor RCPs.

## 5. Conclusions

In conclusion, the rennet coagulation properties of milk significantly affect cheese yield.

Indeed, the vat milk with optimal rennet coagulation properties was characterised by higher values of cheese yield than milk with poor RCPs (8.79 vs. 8.08 kg/100 kg). This higher cheese yield ability was associated with a higher content of calcium (122.51 vs. 110.10 mg/100 g), phosphorus (95.02 vs. 88.78 mg/100 g), and citric acid (181.10 vs. 166.47 mg/100 g). Indeed, phosphate and citrate were found to be positively correlated with milk pH values, with positive repercussions on the primary phase of coagulation and leading to a reduction in milk clotting time.

Moreover, the content of calcium phosphate was positively associated with the secondary phase of the rennet coagulation process, resulting in a lower curd firming time and higher curd firmness for vat milk with optimal and sub-optimal rennet coagulation properties compared to vat milk with poor rennet coagulation properties. The last two parameters positively affect curd strength to cut and curd strength to compression, with positive repercussions on the efficiency of the cheese-making process. In fact, cheese-making using vat milk with optimal and sub-optimal rennet coagulation properties, compared to using milk with poor rennet coagulation properties, was characterised by lower fat losses.

## Figures and Tables

**Table 1:** A schematic summary of the experimental design: 2 years, in 10 cheese factories, 20 trials, 1 trial per cheese factory per year, 60 cheese-making processes, and 3 cheese-making processes per cheese factory per trial.

Operation	Number	Description
Duration of experimental design	2 years	Two consecutive years
Cheese factories involved	10 cheese factories	Located in the Parma and Reggio Emilia provinces
Trials performed	20 experimental trials	One trial per cheese factory per year
Cheese-making processes	60 cheese-making processes	Three cheese-making processes per cheese factory per trial: optimal, sub-optimal, and poor rennet coagulation properties

**Table 2:** A schematic summary of the classification of the rennet coagulation classes: optimal, sub-optimal, and poor.

Clotting Time (Minutes)	Curd Firmness (Millimetres)	Lactodynamograms Type	Rennet Coagulation Classes
Less than 5	Between 0 to 100	DD	Poor
Between 5 and 8	Less than 30	C	Suboptimal
Between 5 and 8	Between 30 to 53	AB	Optimal
Between 5 and 8	Between 54 to 100	CC	Suboptimal
Between 8 and 11	Less than 30	C	Suboptimal
Between 8 and 11	Between 30 to 40	AB	Optimal
Between 8 and 11	Between 40 to 100	AD	Suboptimal
Between 11 and 17.30	Less than 19	AE	Suboptimal
Between 11 and 17.30	Between 19 to 40	A	Optimal
Between 11 and 17.30	Between 40 to 100	B	Optimal
Between 17.30 and 20	Less than 19	E	Poor
Between 17.30 and 20	Between 19 to 40	EA	Suboptimal
Between 17.30 and 20	Between 40 to 100	B	Optimal
Between 20 and 22	Less than 19	E	Poor
Between 20 and 22	Between 19 to 100	EA	Suboptimal
Between 22 and 26	Less than 100	E	Poor
Between 26 and 28	Less than 4	EF	Poor
Between 26 and 28	Between 4 to 100	E	Poor
Between 28 and 30	Less than 100	EF	Poor

**Table 3:** Chemical composition and somatic cell count of whole milk (W-milk) with optimal, sub-optimal, and poor rennet coagulation properties (least square mean values ± standard error).

		Optimal *n* ^1^ = 20		Sub-Optimal *n* ^1^ = 20		Poor *n* ^1^ = 20			
Parameters	Measure Units	Mean		Mean		Mean		SE ^2^	*p* ^3^
Fat	g/100 g	3.64	b	3.68	b	3.58	a	0.03	*
Crude protein	g/100 g	3.25		3.24		3.24		0.02	NS
Casein	g/100 g	2.51		2.50		2.50		0.02	NS
Somatic cells	10^3^ cells/mL	249		250		243		32	NS
Total bacterial count	10^3^ CFU/mL	49		54		36		16	NS

^1^ Number of bulk milk samples. ^2^ Standard error of mean. ^3^ Significance of differences: NS, *p* > 0.05; * *p* ≤ 0.05; a, b different for *p* ≤ 0.05.

**Table 4:** Chemical composition, mineral contents, and somatic cell count of vat milk (V-milk) with optimal, sub-optimal, and poor rennet coagulation properties (least square mean values ± standard error).

		Optimal *n* ^1^ = 20		Sub-Optimal *n* ^1^ = 20		Poor *n* ^1^ = 20			
Parameters	Measure Units	Mean		Mean		Mean		SE ^2^	*p* ^3^
Lactose	g/100 g	5.00	b	5.04	b	4.91	a	0.03	***
Fat	g/100 g	2.75		2.72		2.73		0.03	NS
Crude protein	g/100 g	3.28		3.27		3.26		0.04	NS
Whey protein	g/100 g	0.75		0.75		0.74		0.01	NS
Casein	g/100 g	2.53		2.52		2.52		0.02	NS
Casein number	%	77.13		77.06		77.30		0.16	NS
NPN × 6.38	g/100 g	0.17		0.17		0.16		0.01	NS
Fat-to-casein ratio	Value	1.09		1.08		1.08		0.02	NS
Calcium	mg/100 g	122.51	c	115.73	b	110.10	a	0.94	**
Phosphorus	mg/100 g	95.02	c	91.14	b	88.78	a	0.72	***
Magnesium	mg/100 g	11.97	b	10.86	b	10.03	a	0.11	*
Citric acid	mg/100 g	181.10	c	172.13	b	166.47	a	1.71	*
Chloride	mg/100 g	90.18	a	91.06	a	97.94	b	1.18	*
Somatic cell count	10^3^ cells/mL	172	a	157	a	220	b	11	*

^1^ Number of bulk milk samples. ^2^ Standard error of mean. ^3^ Significance of differences: NS, *p* > 0.05; * *p* ≤ 0.05; ** *p* ≤ 0.01; *** *p* ≤ 0.001; a, b, c different for *p* ≤ 0.05.

**Table 5:** Physico-chemical characteristics, rennet coagulation properties, and rheological parameters of vat milk (V-milk) with optimal, sub-optimal, and poor rennet coagulation properties (least square mean values ± standard error).

		Optimal *n* ^1^ = 20		Sub-Optimal *n* ^1^ = 20		Poor *n* ^1^ = 20			
Parameters	Measure Units	Mean		Mean		Mean		SE ^2^	*p* ^3^
Titratable acidity	°SH/50 mL	3.44	c	3.30	b	3.08	a	0.02	***
pH	Value	6.68	a	6.70	b	6.72	b	0.01	*
Clotting time	minutes	15.50	a	18.53	b	21.18	c	0.27	***
Curd firming time	minutes	6.24	a	9.02	b	10.92	c	0.87	*
Curd firmness	millimetres	35.73	c	28.82	b	17.02	a	1.22	***
Strength to cut	grams	68.97	b	64.43	b	44.38	a	2.86	**
Strength to compression	grams	31.48	b	30.49	b	25.70	a	1.12	**

^1^ Number of bulk milk samples. ^2^ Standard error of mean. ^3^ Significance of differences: * *p* ≤ 0.05; ** *p* ≤ 0.01; *** *p* ≤ 0.001; a, b, c different for *p* ≤ 0.05.

**Table 6:** Actual cheese yield values and cheese-making loss parameters of vat milk (V-milk) with optimal, sub-optimal, and poor rennet coagulation properties (least square mean values ± standard error).

		Optimal *n* ^1^ = 20		Sub-Optimal *n* ^1^ = 20		Poor *n* ^1^ = 20			
Parameters	Measure Units	Mean		Mean		Mean		SE ^2^	*p* ^3^
Actual cheese yield	Kg/100 kg	8.79	b	8.56	ab	8.08	a	0.10	**
Protein losses	%	26.90		26.77		26.52		0.16	NS
Fat losses	%	14.23	a	15.48	ab	16.72	b	0.58	*
Calcium losses	%	35.12		35.99		35.48		0.48	NS
Phosphorus losses	%	48.40		48.95		49.44		0.38	NS
Magnesium losses	%	74.88		75.60		76.13		1.28	NS

^1^ Number of bulk milk samples. ^2^ Standard error of mean. ^3^ Significance of differences: NS, *p* > 0.05; * *p* ≤ 0.05; ** *p* ≤ 0.01; a, b different for *p* ≤ 0.05.

**Table 7:** Pearson correlation coefficients of milk mineral contents with milk rennet coagulation properties and rheological parameters (60 samples analysed).

	Calcium		Phosphorus		Citric Acid	
	r ^1^	*p* ^2^	r ^1^	*p* ^2^	r ^1^	*p* ^2^
Clotting time	−0.378	**	−0.376	**	−0.479	**
Curd firming time	−0.481	**	−0.451	**	−0.237	NS
Curd firmness	0.542	***	0.672	*	0.341	*
Strength to cut	0.512	***	0.609	***	0.080	NS
Strength to compression	0.511	***	0.624	***	0.302	NS

^1^ Pearson correlation coefficient. ^2^ Significance of coefficients compared to zero: NS, *p* > 0.05; * *p* ≤ 0.05; ** *p* ≤ 0.01; *** *p* ≤ 0.001.

**Table 8:** Pearson correlation coefficients of rheological parameters with milk rennet coagulation properties and rheological parameters (60 samples analysed).

	Strength to Cut		Strength to Compression	
	r ^1^	*p* ^2^	r ^1^	*p* ^2^
Clotting time	−0.218	NS	−0.371	NS
Curd firming time	−0.527	***	−0.672	***
Curd firmness	0.450	**	0.656	***
Strength to cut	-	-	0.549	***
Strength to compression	0.549	***	-	-

^1^ Pearson correlation coefficient. ^2^ Significance of coefficients compared to zero: NS, *p* > 0.05; ** *p* ≤ 0.01; *** *p* ≤ 0.001.

**Table 9:** Pearson correlations coefficients between actual cheese yield and protein and fat losses and milk rennet coagulation properties and rheological parameters (60 samples analysed).

	Actual Cheese Yield		Protein Losses		Fat Losses	
	r ^1^	*p* ^2^	r ^1^	*p* ^2^	r ^1^	*p* ^2^
Clotting time	−0.089	NS	0.101	NS	−0.063	NS
Curd firming time	−0.505	**	−0.099	NS	0.578	***
Curd firmness	0.456	**	−0.054	NS	−0.486	*
Strength to cut	0.523	***	−0.068	NS	−0.129	NS
Strength to compression	0.637	***	0.037	NS	−0.440	*
Actual cheese yield	-	-	0.114	NS	−0.355	*

^1^ Pearson correlation coefficient. ^2^ Significance of coefficients compared to zero: NS, *p* > 0.05; * *p* ≤ 0.05; ** *p* ≤ 0.01; *** *p* ≤ 0.001.

## Data Availability

The datasets presented in this article are not readily available because the data are part of an ongoing study. Requests to access the datasets should be directed to the corresponding Author Piero Franceschi at piero.franceschi@unipr.it.
